# 16S rRNA gene sequencing and MALDI TOF mass spectroscopy identification of *Leuconostoc mesenteroides* isolated from Algerian raw camel milk

**DOI:** 10.1186/s43141-023-00500-1

**Published:** 2023-05-01

**Authors:** Hanane Fatma Chentouf, Fouzia Rahli, Zineb Benmechernene, Jorge Barros-Velazquez

**Affiliations:** 1 Laboratory of Applied Microbiology, Department of Biology, Faculty of Natural Sciences and Life, University of Oran, 1 Ahmed Ben Bella, 31100 Oran, Algeria; 2 Higher School of Biological Sciences of Oran, Oran, Algeria; 3 Department of Analytical Chemistry, Nutrition and Food Science, Faculty of Veterinary Sciences, University of Santiago, 27002 Lugo, Spain

**Keywords:** 16S rRNA gene sequencing, MALDI-TOF MS, *Leuconostoc mesenteroides*, Algerian raw camel milk

## Abstract

**Background:**

Eighty-three strains of *Leuconostoc mesenteroides* were isolated from Algerian raw camel milk. Based on morphological, biochemical, and physiological characters tests, strains were identified as *Ln. mesenteroides* subsp. *mesenteroides.* Seven strains had a remarkable antagonistic and probiotic characterization. The present study aims at identifying these strains by means of 16 s rRNA gene sequencing and Matrix-assisted laser desorption/ionization time-of-flight mass spectrometry (MALDI-TOF MS), extending phenotypic and genotypic studies done previously.

**Results:**

The phyloproteomic dendrograms of the studied strains based on MALDI-TOF MS provided the same identification with more intraspecific information from the 16S rRNA gene sequencing based on phylogenetic analysis. The latter were in agreement with the previous biochemical/physiological identification, the seven isolated strains were *Ln. mesenteroides* subsp. *mesenteroides.*

**Conclusions:**

Remarkably, MALDI-TOF MS fingerprinting was found to be effective enough as 16S rRNA gene sequencing identification, allowing faster and more reliable analysis than biochemical/physiological methods.

## Background

In Algeria, as in many countries with arid and desert ecosystems, camels (*Camelus dromedarius*), because of their great adaptation to hostile climatic conditions, play an important role in the life of these communities by providing meat, milk, and transport [1, 2]

Camel milk is a high-quality nutritious food due to its richness in proteins, carbohydrates, lipids, vitamins, and mineral salts [3, 4], and high bacterial diversity, suggesting a potential resource for the food industry [5] with antimicrobial potential, antiviral and protective activities against pathogenic bacteria [6]. Due to that, it has experienced renewed interest in recent years. Although it has been the subject of multiple works from all over the world especially in Algeria [7].

Camel milk microflora’s recent study showed its composition of four phyla; Firmicutes (64.7%), Proteobacteria (30%), Actinobacteria (4.9%), and Bacteroidetes (0.4%) [5]. The higher numbers were attributed to lactic acid bacteria (LAB) and leuconostocs have often been found as the major genus in camel milk [8–10].

The taxonomic characterization of the 29 *Leuconostoc* species is still controversial and the methods applied including cultivation, phenotypic/biochemical, and non–specific molecular methods could lead to misidentifications [11], especially in sub-level to differentiate among the *Leuconostoc mesenteroides* subspecies. To date, five subspecies are included *Ln. mesenteroides* subsp*. mesenteroides*,* Ln. mesenteroides* subsp*. cremoris*,* Ln. mesenteroides* subsp*. dextranicum*,* Ln. mesenteroides* subsp*. Suionicum*, and *Ln. mesenteroides* subsp. *Jonggajibkimchii* (lpsn.dsmz.de/genus/leuconostoc). They have been known to play important roles in the production of various fermented foods, the food industry, and food preservation [12–15].

Using physiological, serological, and biochemical methods, it typically takes a lot of work, resources, and time to identify microorganisms (such bacteria, yeasts, and molds). In addition to these techniques, several molecular (nucleic acid-based) approaches have been developed. Most of the genotypic identification methods are mainly based on the polymorphism of the 16S rRNA gene sequences. Sequencing of the 16S rRNA gene has been a reference method for species-level identification of various groups of bacteria [1]. Polymerase chain reaction tests developed with primers and probes directed toward the identification of gene sequences encoding 16S ribosomal RNA can identify the presence of DNA from most bacterial species [2]. Among the conserved genes that encode rRNA, 16S rRNA genes are considered as a standard for microbial identification and taxonomic classification [16]. Polymerase chain reaction (PCR) targeting either partial or whole 16S rRNA gene has been applied for the identification of several *Leuconostoc* spp. from Algerian camel milk [10, 17, 18].

Although the reliability of the applied genomic methods, such as 16S rRNA gene sequence analysis, is high, they cannot provide quick results. However, new technologies for the accurate and rapid identification of bacteria are essential in various fields of applied microbiology. Mass spectrometry is an alternative solution for identifying and strain typing [19]. MALDI TOF technique is a bacterial chemotaxonomic method with strong potential in bacterial species differentiation and has been proven to be effective [20], this method can examine the mass-to-charge ratio of a wide range of biomolecules, including peptides and proteins [21]. it can be considered a fast, accurate, and low-cost method for the identification of Gram-positive bacteria such as LAB [22]. The ribosomal proteins in particular are the most reliable biomarkers for identifying bacteria because of their abundance and moderate hydrophobicity, they are well-suited for effective ionization [23]. Thus, using this technique, an adequate amount of stable mass signals for the ribosomal protein peptides typically 2000 to 20,000 Da can be obtained. The mass signals are used to produce profile spectra, which comprise a series of peaks that are conserved at the genus, species, and even subspecies level [24]

The present study aims at identifying *Leuconostoc mesenteroides* collected from Algerian raw camel milk, by means of 16 s rRNA sequencing and Matrix-assisted laser desorption ∕ ionization time-of-flight mass spectrometry (MALDI-TOF MS), extending phenotypic and genotypic studies done previously [25] by comparing these two techniques with the previous classical methods in accuracy, cost, efficacity.

## Methods

### Strains origin and culture conditions

The strains were isolated from 12 raw camel milk samples that were obtained from different Algerian arid zones. The milk was collected in sterile glass bottles; stored at 4 to 6 °C before analysis. The samples were analyzed within 12 to 30 h of collection.

Eighty-three strains of *Leuconostoc mesenteroides* were isolated using a selective medium, MRS (pH 6.8) supplemented with 30 µg/ml of vancomycin. They were identified by phenotypic and biochemical methods. We retained seven strains (*Ln*C12, *Ln*C21, *Ln*C23, *Ln*C26, *Ln*C28, *Ln*C29, and *Ln*C33) that showed remarkable inhibitory activity against *Listeria* spp. including *L. monocytogenes*. The strains were subcultured and enriched in MRS broth for 6 h at 37 °C. (Supplementary details on camel’s milk, collected samples, or phenotypical, biochemical, and physiological tests could be found on Chentouf and Benmechernene, 2013 research paper [25].

Reference strains used for genetic identification are from different sources; a standard collection of Spanish cultures and a standard collection of cultures from the University of Ghent (Belgium). Reference strains are shown in Table 1.

**Table 1:** Reference strains used in phylogenetic and proteomic identification

Strains	Source	Origin
*Leuconostoc mesenteroides* subsp. *mesenteroides*	CECT 219	Fermented olive
*Leuconostoc mesenteroides*	LMG 6908	ND
*Leuconostoc pseudomesenteroides*	CECT 4027	Juice
*Leuconostoc pseudomesenteroides*	LMG 11,482	ND
*Leuconostoc carnosum*	CECT 4024	Beef
*Lactococcus lactis* subsp. *lactis*	CECT 4432	Cow milk
*Lactococcus lactis* subsp. *cremoris*	LMG 1363	Cow milk

*CECT* Spanish-type culture collection, *LMG* collection-type cultures of Ghent University (Belgium), *ND* not determined

### Bacterial identification and phylogenetic analysis based on 16S rRNA gene sequencing

In order to isolate genomic DNA, the protocol of Fernández-No et al. (2013) [26] was used. The PCR amplification was carried out with p8FPL/p806R universal primer pair: p8FPL (5′-AGTTTGATCCTGGCTCAG-3′) and p806R (5′-GGACTACCAGGGTATCTAAT-3′). DNA extraction was performed using the DNeasy Kit (Invitrogen).

The PCR reaction was performed in a “My Cycler” thermal cycler (BioRad Laboratories, Hercules, USA). The cycling program was as follows: template DNA was initially denatured at 94 °C for 7 min. Thereafter, products were amplified by 35 cycles. Each cycle consists of denaturation for 60 s at 94 °C, primer annealing for 60 s at 55 °C, and extension for 60 s at 72 °C. The last cycle was followed by a final extension at 72 °C for 15 min. The PCR was performed as described by Bohme et al. (2011) [27]. PCR products were resolved by electrophoresis, using a 2.5% agarose gel in TAE buffer (Tris- Acetate-EDTA).

The 16S rRNA gene isolates were subjected to sequencing using both primers used for PCR (Forward (p8FPL) and Reverse (p806R). Sequencing reactions were analyzed in an automated sequencing system (ABI 3730XL DNA Analyzer, Applied Biosystems) with the POP-7 system. The gene sequences were analyzed with Chromas software (Griffith University, Queensland, Australia) and aligned using Clustal X software [28, 29]. These sequences were identified by searching for sequence homology between published reference sequences using the BLAST (National Center for Biotechnology Information (NCBI), http://blast.ncbi.nlm.nih.gov/) [30]. Homologies with 99–100% a strain type were considered as excellent identifications.

Phylogenetic and molecular analyses were performed with MEGA 5.0 software [31]. Phylogenetic clustering and tree-based phylogeny construction were performed using the neighbor-joining method [28] using the “Bootstrap method” as a test of phylogeny [32, 33]. Evolutionary divergence was estimated using MEGA 5.0 software [33].

### Identification of *Leuconostoc* strains by the MALDI-TOF MS

According to the method described by Böhme et al. (2010a, 2010b) [34, 35], bacterial isolates were prepared for MALDI-TOF MS. which was based on the lysis of bacterial cells with an aqueous trifluoroacetic acid (TFA) and acetonitrile (ACN) combination (Acros Organics, Morris Plains, NJ) and the subsequent mixing of each sample with a matrix solution made up of cyano-4-hydroxycinnamic acid (-CHCA), acetonitrile, and TFA to extract the cell proteins.

With the use of the parameters described in Benmechernene et al. (2014) [36], mass spectra were produced using a voyager mass spectrometer (Applied Biosystems, Foster City, CA). There were two extractions for each strain and double measurements of the two extracts, yielding a total of four spectra for each bacterial strain. Data Explorer software (version 4.0) (Applied Biosystems) was used to analyze the mass spectra for baseline correction and noise filtering after they had been externally calibrated using a mixture of 1 pmol/l oxidized B-chain insulin and 1 pmol/l bovine insulin (Sigma-Aldrich, St. Louis, MO).

Mass spectra were meticulously analyzed with Data Explorer software (version 4.0). Data listings with m/z values have been extracted from mass spectrometry data including signals with relative intensities greater than 2%. The mass peak lists obtained were analyzed and compared using peaks that spanned from 2000 to 10,000 Da in mass because of the reproducibility of the spectral profile in this mass range.

The mass lists of all studied strains have been clustered using the SPECLUST web interface http://bioinfo.thep.lu.se/speclust.html [37] as described by Quintela-Baluja et al. (2013) [38].

Finally, phyloproteomic clustering was confirmed by mass list analysis utilizing Statgraphics Plus software (version 5.1).

## Results

### Genetic identification of Leuconostoc strains by 16S rRNA gene sequencing

Using the previously described universal 16S rRNA primer pair, all bacterial strains were submitted to genetic analysis based on a 1500-bp fragment of the 16S rRNA gene. The obtained sequences of each sample were compared against strains placed in the NCBI GenBank database (www.ncbi.nlm.nih.gov) using the BLAST tool. All the seven strains (*Ln*C12, *Ln*C21, *Ln*C23, *Ln*C26, *Ln*C28, *Ln*C29, *Ln*C33) were successfully identified as *Leuconostoc mesenteroides.*

**Fig. 1:**
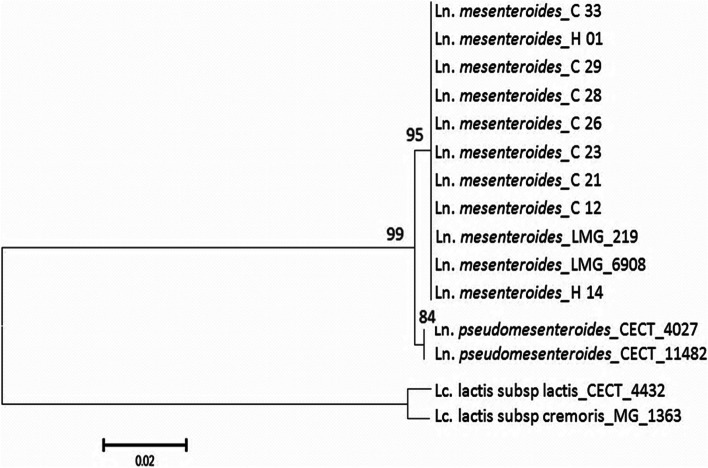
Phylogenetic dendrograms of the studied strains based on 16S rRNA gene sequencing

In order to construct the phylogenetic tree, the genus *Lactococcus*, which belongs to the group of lactic acid bacteria, was chosen as an outgroup. The genus *Ln. pseudomesenteroides* is quite phylogenetically close to *Leuconostoc* (Fig. 1).

### MALDI-TOF MS fingerprinting and phyloproteomic clustering

Four spectra were obtained for each strain to ensure the reproducibility of these results [39]. The mass lists for each of the 28 strains analyzed were constructed using the SPECLUST application by determining the arithmetic means for m/z values and the standard deviation was less than 5 Da in the mass range of 2000–10,000 Da. The mass lists include 72 mass peaks that were generated for 7 *Leuconostoc* strains with two *Lactococcus* strains classified as an outgroup. While 54 peaks were observed in *Leuconostoc* strains, 18 peaks were specific to the *Lactococcus* genus and only two peaks were shared by the two genera.

It should be noted that peaks at m/z 6242 Da and m/z 5118 Da were present in all *Leuconostoc* species. These peaks are specific for the genus *Leuconostoc* (Table 2).

**Table 2:** Mass peaks of specific species of *Ln. carnosum*, *Ln. pseudomesenteroides*, and *Ln. mesenteroides* [36]

Microbial species		
***Ln. carnosum***	*Ln. pseudomesenteroides*	*Ln. mesenteroides*
4424		
5123	4388	
5866	5104	6242
6368	6225	5118
7065	7942	
7601		

**Fig. 2:**
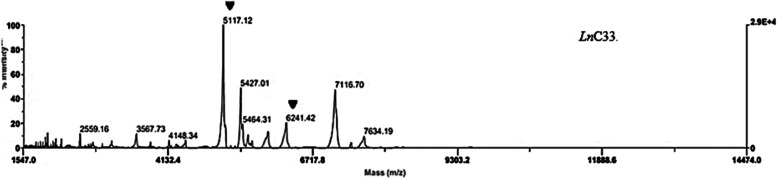
MALDI-TOF MS spectra of the *Ln*C33 *Leuconostoc mesenteroides* strain isolated from camel milk*.* Specific genus peaks marked as (▼) are present in other *Leuconostoc* strains

**Fig. 3:**
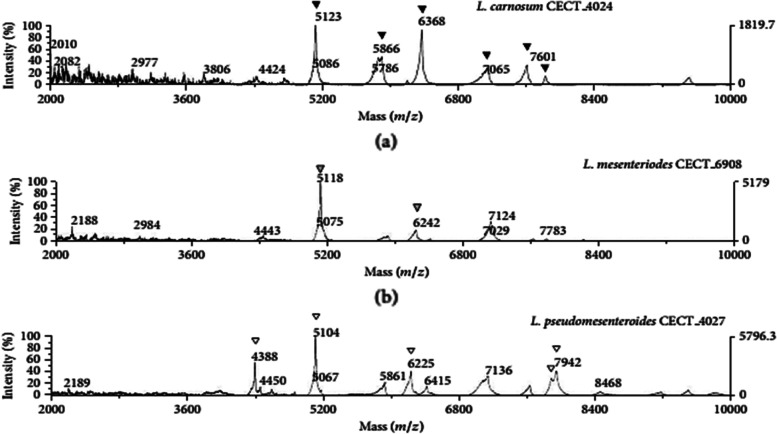
MALDI-TOF MS spectra of *Ln. carnosum*, *Ln. mesenteroides,* and *Ln. pseudomesenteroide* reference strains. Common peaks to each strain are indicated by (▼) [36]

The MALDI-TOF MS spectral profile (fingerprint) revealed good results for all the analyzed strains. The mass lists include 62 masses peaks that were generated from 7 *Leuconostoc* strains (data not shown). Thus, from the 54 peaks present in the genus *Leuconostoc*, 17 were present in more than 90% of the analyzed samples. Among them, 2 peaks (5118 et 6242 Da) were observed in common (Fig. 2) comparing with MALDI-TOF MS spectra of *Ln. carnosum*, *Ln. mesenteroides*, and *Ln. pseudomesenteroide* reference strains (Fig. 3).

**Fig. 4:**
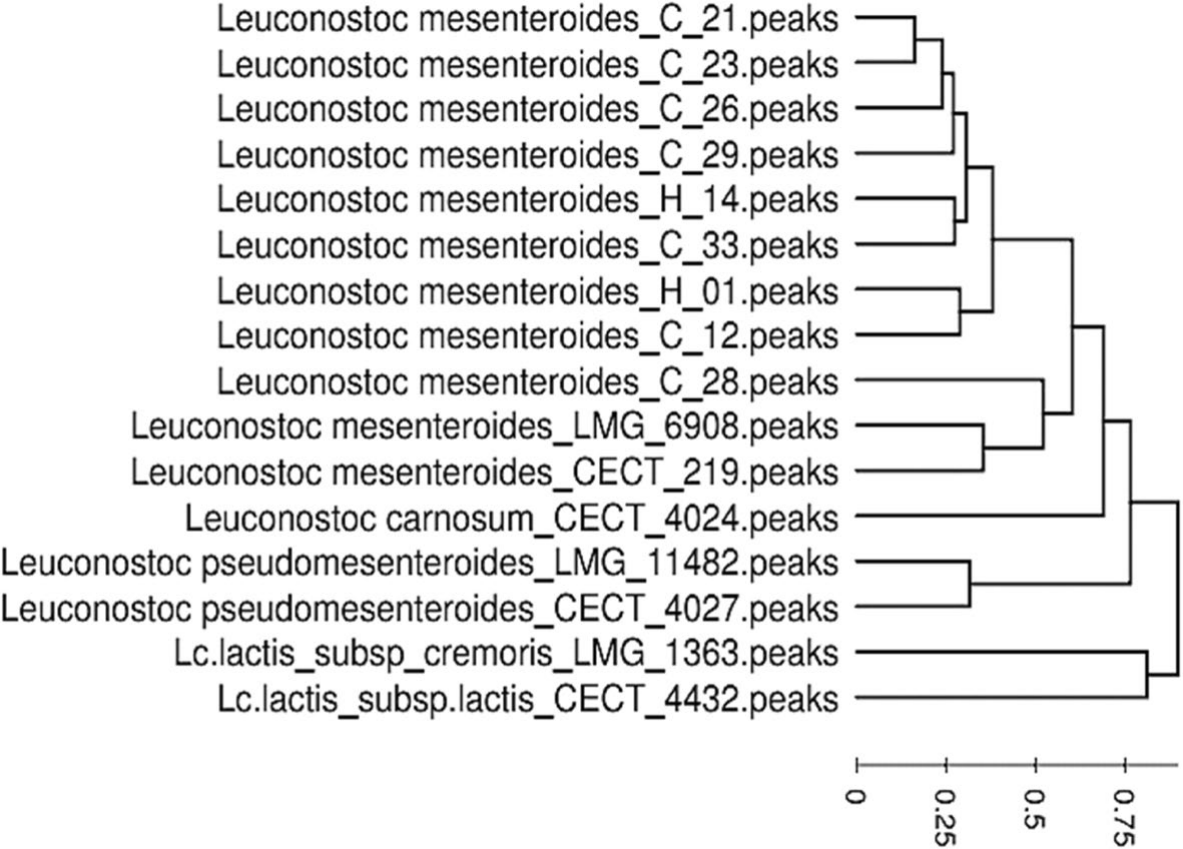
Phyloproteomic dendrograms of the studied strains based on MALDI-TOF MS fingerprinting

Furthermore, the phyloproteomic dendrograms of the studied strains based on MALDI-TOF MS fingerprinting (Fig. 4) provided more intraspecific information from the 16S rRNA gene sequencing based on phylogenetic analysis (Fig. 1), allowing precise discrimination among all *Leuconostoc* species and with the out-group strains (*L. lactis*). Clearly, all the studied strains belonging to the same species or subspecies were clustered consistently as separate groups. Thus, the phyloproteomic clustering showed a clear differentiation of *Leuconostoc* species. All the isolates are set together with the *Ln. mesenteroides* LMG 6908 and *Ln. mesenteroides* subsp *mesenetroides* CECT 219 reference strains, however, the other reference strains were out-grouped.

## Discussions

Raw camel milk is a good source of dairy LAB strains, including *Leuconostoc* spp. [9, 40, 41]. Several methods have been developed for identification of *Leuconostoc*, which include Phenotypical, biochemical characterization, and different genotypical methods such as 16S rRNA gene sequencing, RAPD fingerprinting, Pulse field gel electrophoresis (PFGE), chromosomal DNA restriction analysis and multiple molecular markers sequencing [42]. In recent years, MALDITOF MS was found to be very fast and effective as molecular microorganism identification method [43, 44].

A number of 83 strains of *Leuconostoc* were isolated, earlier, from raw camel milk in arid and semi-arid zones of Algeria. Based on morphological, biochemical and physiological tests, these strains were identified as *Leuconostoc mesneteroides* subsp. *mesenteroides.* Among them, seven strains had an exceptional antagonistic and probiotic properties. In this work, these strains (*Ln*C12, *Ln*C21, *Ln*C23, *Ln*C26, *Ln*C28, *Ln*C29, *Ln*C33) were identified and typed by molecular methods.

The phylogenetic tree based on 16S rRNA gene sequencing permitted a clear differentiation between and within *Leuconostoc* and *Lactococcus*. Thus, phylogenetic analysis indicated that all strains isolated from raw camel milk were grouped into a common cluster with reference strains *Ln. mesenteroides* subsp. *mesenteroides* (LMG 219) and *Ln. mesenteroides* (LMG 6908). This confirmed the identity of these strains as *Ln. mesenteroides* subsp. *mesenteroides.* However, *Ln. pseudomesenteroides* (CECT 11,482) and *Ln. pseudomesenteroides* (CECT 4027) were grouped together but were not in the same cluster as *Ln. mesenteroides* strains. *Lactococcus* strains are grouped into two distinct subgroups corresponding to *Lactococcus lactis* subsp. *cremoris* (MG 1363) and *L. lactis* subsp*. lactis* (CECT 4432). These two subgroups were separated by a short distance due to their genetic similarity to *Ln. mesenteroides* and *Ln. pseudomesenteroides*. The sequencing of 16S rRNA for the studied strains confirmed phenotypical and genotypical identification results. The strains *Ln*C12, *Ln*C21, *Ln*C23, *Ln*C26, *Ln*C28, *Ln*C29, *Ln*C33 belonged to *Leuconostoc mesenteroides* subsp. *mesenteroides.* A study on genomic identification of *Leuconostoc* spp. isolated from Algerian raw camel milk was carried out by Benmechernene et al. (2014) [36] and Mokdad et al. (2020) [18] has been made which showed similar results.

However, the 16S rRNA gene has limitations in differentiating among closely related species and subspecies needing additional techniques, such as the Matrix-assisted laser desorption ∕ ionization time-of-flight mass spectrometry (MALDI-TOF MS) [45].

The objective of the proteomic analysis was to quickly and easily extract low molecular weight soluble proteins from entire bacterial cells. It relies on the detection of mass-to-charge ratio abbreviated as m/z of ribosomal proteins of the bacteria, which helps to provide a unique mass spectrum of the bacteria within a short period of time [46]. One of the main benefits of this extraction method is that it allows appropriate contact time between bacterial cells and lysis reagents, leading to good cellular protein extraction and high reliable spectra that allow inter- and intraspecies differentiation within a mass range of 2000–10,000 Da [47].

The identification with MALDI-TOF MS is provided by comparing the spectrum with the spectra of reference strains based on the closest match [48], there are few studies on molecular identification using MALDI-TOF MS of *Leuconostoc mesenteroides* and the most similar study to ours in terms of bacterial provenance and all other conditions is that of Benmechernene et al. (2014) [36].

All bacterial strains that were submitted to the MALDI-TOF MS assay generated a spectrum that permitted all *Leuconostoc* strains to be differentiated at to the species level, although to the subspecies level. The two peaks were shared by all the analyzed strains with the slight difference compared with those found by Benmechernene et al. (2014) [36] such as the highest common intensity peak that appeared at m/z 5117.12 Da, and the second at m/z 6241.42 Da instead of 5118 and 6242, respectively; this may be caused by bacterial response to stress and environmental changes, including storage and handling [36].

Exceptionally, fingerprinting revealed different results; the 7110 Da mass peak was observed at several *Leuconostoc* strains and it was not considered as *Leuconostoc mesenteroides*-specific mass peak. Comparing our mass lists peaks with *Leuconostoc mesenteroides* identified by Benmechernene et al. (2014) [36]; three peaks were noticed in common (5556, 7113, and 7626 Da) these peaks could serve as biomarker peaks in future analyses considering that the technique takes into account the biomarker ion peaks in a spectrum and relies on the characteristic mass profile obtained by a set of ion peaks, which represents a "fingerprint" of the bacteria [49].

Therefore, phyloproteomic analysis allowed to group *Ln. mesenteroides* strains into different subgroups, while the phylogenetic proximity of these strains did not allow such differentiation [50]. The use of MALDI-TOF MS for the characterization of lactic acid bacteria was carried out by De Bruyne (2011) [50] and Benmechernene et al. (2014) [36] regarding the proteomic identification of *Leuconostoc* from food sources and Algerian raw camel milk, respectively.

Molecular identification of all seven strains concorded with the phenotypic/biochemical identification done previously [25] as *Leuconostoc mesenteroides* subsp. *mesenteroides.*

The application of MALDI TOF mass spectrometry fingerprinting has been successfully applied to this bacterial group and has proven to be a simple, fast, and inexpensive complementary method for bacterial identification to the species and subspecies level, unlike phenotypic/biochemical methods which were very long in fact, however, MALDI TOF MS helps in avoiding unworthy identification of clone of the same strain that could be wrong in classical methods [20].

Researchers consider it as the most reliable identification technique taking intact cells or crude protein extracts are directly analyzed resulting in a mass spectral profile, which is characteristic for the considered organism [16].

## Conclusion

In conclusion, this study employed nucleotide sequencing, phylogenetic analysis, and MALDI-TOF mass fingerprinting, in the identification of *Leuconostoc* spp. isolated from Algerian raw camel milk and it has provided more genetic characterization for the species. Additionally, the identification was necessary for further studies like using *Leuconostoc mesenteroides* as a starter in various fermenting food.

## Data Availability

The data and materials that support the findings of this study are available on request from the corresponding author. Supplementary data to this article could be found at https://doi.org/10.5897/AJMR2013.5753

## Abbreviation

ACN: Acetonitrile
CECT: Colección Española de Cultivos Tipo (Spanish Type Culture Collection)
DNA: Deoxyribonucleic acid
L.: Listeria
LAB: Lactic acid bacteria
LMG: Laboratory of Microbiology of Ghent University (Belgium)
Ln.: Leuconostoc
MALDI-TOF MS: Matrix-assisted laser desorption/ionization time-of-flight mass spectrometry
MRS: Man Rogosa and Sharpe
ND: Not determined
PCR: Polymerase chain reaction
PFGE: Pulse Field Gel Electrophoresis
RAPD: Randomly amplified polymorphic DNA
rRNA: Ribosomal ribonucleic acid
subsp.: Subspecies
TAE: Tris-acetate-EDTA
TFA: Aqueous trifluoroacetic acid
α-CHCA: α-cyano-4-hydroxycinnamic acid
